# Peroperative Intra-Articular Infiltration of Tranexamic Acid and Ropivacaine Cocktail in Patients Undergoing Total Knee Arthroplasty: A Randomized Controlled Trial

**DOI:** 10.7759/cureus.23091

**Published:** 2022-03-12

**Authors:** Harpreet Singh, Kamal Kumar Agarwal, Sangam Tyagi, Prashant Makadia, Bineet Oza, Pranjal Jain, Meet Patel

**Affiliations:** 1 Department of Orthopaedics, Geetanjali Medical College and Hospital, Udaipur, IND

**Keywords:** intra-articular injection, tranexamic acid, ropivacaine, intra-articular cocktail infiltration, total knee arthroplasty

## Abstract

Background

Total knee arthroplasty (TKA) is a procedure that has improved the quality of life of patients with knee arthritis. Postoperative pain and blood loss are the two major drawbacks of TKA which affect patient satisfaction and delay recovery and rehabilitation. Local infiltration analgesia has shown better results in controlling immediate postoperative pain, thus enabling early rehabilitation and mobilization, while local infiltration of antifibrinolytic agents has shown impressive results in controlling blood loss. In this study, we evaluate the effect of a combination of intra-articular infiltration of ropivacaine cocktail along with intra-articular instillation of tranexamic acid in reducing patient-reported postoperative pain and the level of blood loss control after TKA.

Methodology

Patients presenting with high-grade osteoarthritis and undergoing TKA were included and randomly allocated to two groups: one receiving the intra-articular infiltration (group A), and the other not receiving any infiltration (group B). Postoperative pain was measured through the Visual Analog Scale (VAS) every three hours for the first 24 hours, and then at 48 hours and 72 hours postoperatively. The need for additional analgesia, in the form of a slow epidural infusion, in patients experiencing severe postoperative pain was evaluated in both groups. Postoperative blood loss was assessed by measuring total drain output (in mL) and by comparing preoperative and postoperative (at 24 hours) hemoglobin, hematocrit drift, and blood transfusion rates. The duration of the postoperative hospital stay and the time taken to start postoperative knee mobilization exercises and weight-bearing were noted to assess the recovery and rehabilitation of the patients in the two groups.

Results

The study included 42 patients (group A, 22 patients; group B, 20 patients) with 28 knees in each group. Patients with intra-articular infiltration using ropivacaine cocktail with tranexamic acid showed excellent pain control compared to the non-infiltrated patients in the early 48 hours postoperatively. There was a significant drop in postoperative hemoglobin and hematocrit values in the non-infiltrated patients compared to the other group. Further, the intra-articular infiltration-instillation significantly reduced blood loss through the drain, the requirement of postoperative blood transfusions, and the duration of hospital stay.

Conclusions

It can be safely concluded that ropivacaine cocktail and tranexamic acid instillation postoperatively in knee arthroplasty patients is a very useful and effective technique to reduce postoperative pain and blood loss.

## Introduction

Osteoarthritis is a degenerative joint disease that involves the articular cartilage, bone, and surrounding soft tissues, resulting in the obliteration of joint space, osteophyte formation, and deformity [1]. Knee joints show one of the most severe and disabling forms of osteoarthritis. Its incidence is increasing with the increase in the aging population and the prevalence of lifestyle diseases such as obesity [2].

In patients with severe and high-grade knee osteoarthritis, total knee arthroplasty (TKA) is one of the most successful surgical procedures for pain-free, stable, and mobile joints [3]. Around 60% of patients undergoing this surgery experience severe postoperative pain [4]. TKA is also accompanied by a high and formidable risk of bleeding and a subsequent increase in the rates of blood transfusion postoperatively [5].

Many modalities of preoperative, perioperative, and postoperative analgesia, including epidural anesthesia, intrathecal morphine, regional nerve blocks, systemic opioids, local infiltrative analgesia, intra-articular corticosteroids, and intra-articular platelet-rich plasma injections, have been researched and tested to control postoperative pain in patients undergoing TKA [6-10].

Local infiltrative analgesia is an attractive alternative to other modalities by providing analgesia locally at the site of surgical trauma with minimal systemic side effects. Intra-articular infiltrations and injections using various analgesics in patients undergoing TKA surgeries have shown their effectiveness in reducing the requirements for postoperative epidural and systemic analgesia and have led to earlier hospital discharge rates in many studies [6,11].

Ropivacaine is a local anesthetic agent used for spinal or epidural anesthesia and postoperative analgesia. It is generally given intrathecally and as an epidural infusion. Local intra-articular infiltration of ropivacaine in the knee has shown better results in controlling immediate postoperative pain, thus enabling early rehabilitation and mobilization [4,11,12]. Tranexamic acid is an antifibrinolytic agent with a mechanism of action to prevent fibrin degradation. Local infiltration of tranexamic acid has worked well and shown impressive results in controlling blood loss and incidence of blood transfusions compared to the intravenous route of administration. In addition, postoperative drainage blood loss was significantly low in previous studies [13-15].

This study aimed to evaluate the effect of intra-articular infiltration of ropivacaine cocktail along with intra-articular instillation of tranexamic acid for reducing patient-reported postoperative pain and to assess the level of blood loss control postoperatively.

## Materials and methods

After obtaining institutional research board approval and informed patient consent, this study was conducted in the Department of Orthopaedics at Geetanjali Medical College and Hospital, Udaipur, Rajasthan, India, from January 2020 to June 2021 on a sample size of 42 patients. Patients were randomized into two groups based on alternate systematic allocation. Group A included patients receiving the intra-articular infiltration of ropivacaine cocktail and tranexamic acid instillation. Group B included patients not receiving the intra-articular infiltration and instillation.

All adult patients complaining of pain in the knee with difficulty in performing their normal daily activities and radiologically diagnosed with osteoarthritis of the knee joint, grade IV, according to the Kellgren-Lawrence classification system [16], were selected for TKA and included in this study. Patients with a history of allergy to any of the drugs used in the trial, abnormal renal function, blood disorders, or those with hemoglobinopathies were excluded from this study. A detailed history was taken, a systemic examination was performed, and blood samples were sent for all routine preoperative investigations. X-rays of both knees including the anteroposterior view (weight-bearing), lateral view, and a full-length scanogram of both lower limbs were done.

All surgeries were performed by a single surgeon. Surgery was done under spinal anesthesia with an epidural catheter placed without activation. Appropriate bolsters for limb positioning and thigh supports were secured to the table. All patients were operated on with a pneumatic tourniquet. After painting and draping, the limb was exsanguinated with a sterile crepe bandage, and the tourniquet was inflated. An anterior midline incision with a medial parapatellar approach was used to expose the knee. A distal femoral cut was made perpendicular to the mechanical axis using an intramedullary cutting guide. A proximal tibial cut was made using an extramedullary guide. Posterior referencing of the distal femur was done and component size was measured. Anterior, posterior, and chamfer cuts were made. Trial components were then placed, and knee balancing and patellar tracking were checked. The proximal tibia was prepared for component placement. Thorough wound irrigation was done with pulse lavage.

**Figure 1:**
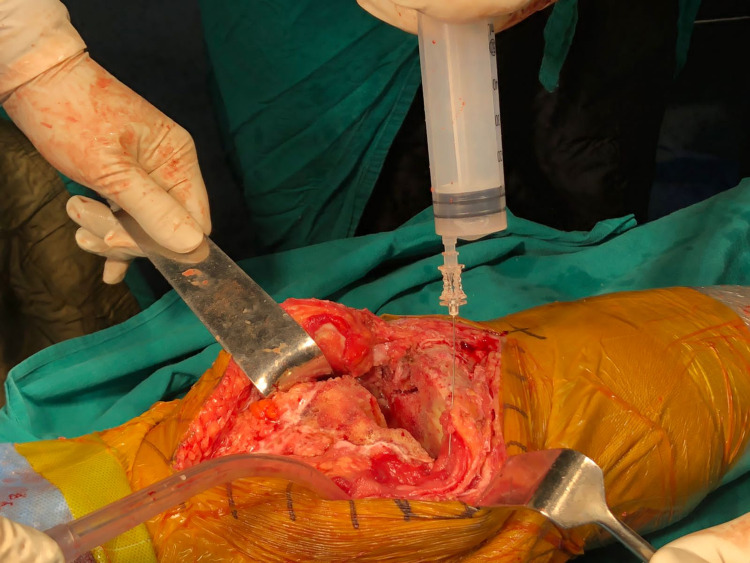
Intra-articular infiltration of ropivacaine cocktail in the medial sleeve.

**Figure 2:**
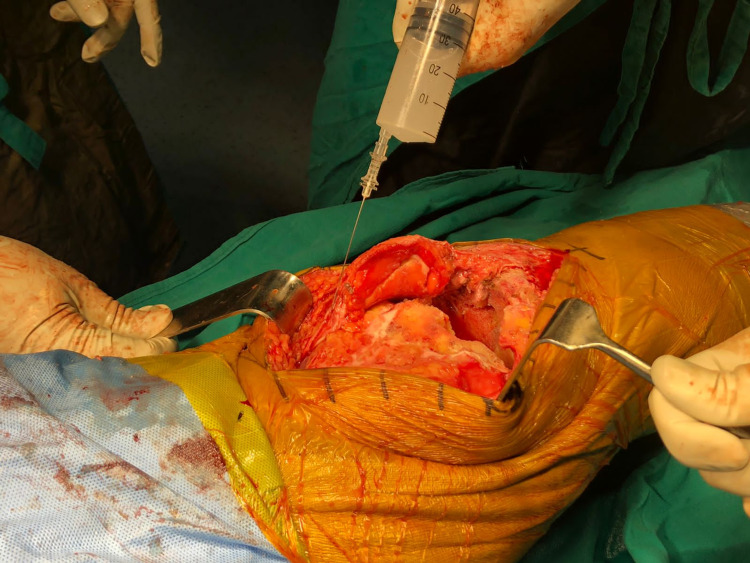
Intra-articular infiltration of ropivacaine cocktail in the lateral sleeve.

**Figure 3:**
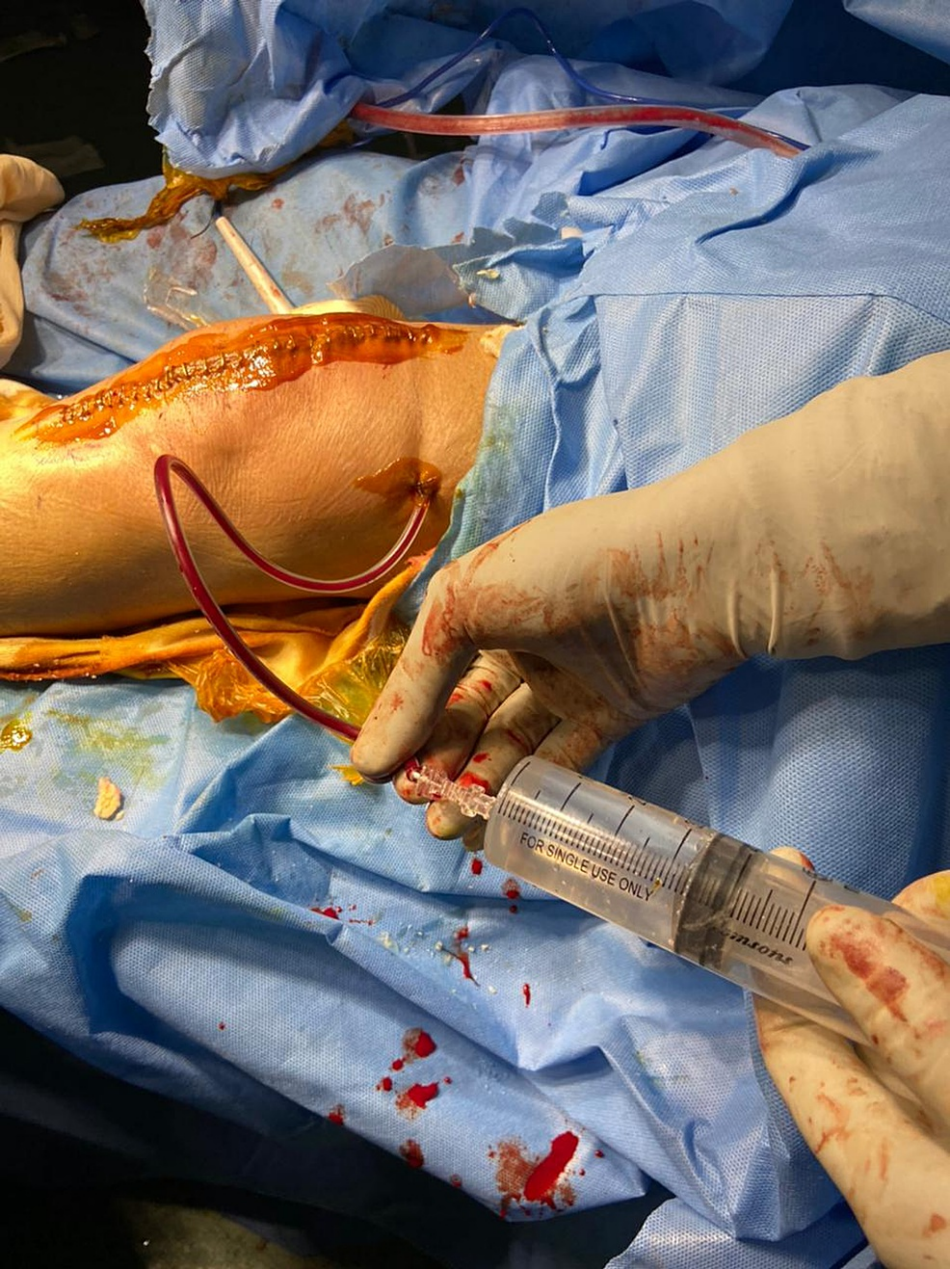
Instillation of tranexamic acid solution through the drain tube.

A cocktail containing 0.2% ropivacaine (50 mL), 40 mg of triamcinolone (1 mL), 30 mg of ketorolac (1 mL) and 40 mg of gentamicin (1 mL) was prepared. The injection was administered using a 20-gauge spinal needle and syringe into the medial sleeve, lateral sleeve and posterior capsule, deep fascia, and the subcutaneous tissue around the skin incision (Figures 1, 2). While infiltrating the posterior areas of the knee, care was taken to avoid injecting into the neurovascular structures. Bone cement was prepared, and the posterior stabilized components were fixed. Patella resurfacing and denervation were done. Complete hemostasis was achieved after deflating the tourniquet, and a suction drain was inserted before wound closure. Instillation of 50 mL solution containing 15 mL of tranexamic Acid (1.5 g), diluted with 35 mL of 0.9% normal saline, was done through the drain tube (Figure 3). A collection bag was then attached to the drain with negative pressure, and the drain was kept clamped for approximately one hour, after which it was opened. An epidural catheter was kept in place without activation. A sterile compression bandage was applied to all patients. Group B patients did not receive any such infiltration-instillation.

For all patients, postoperative thromboprophylaxis was started on the day of the surgery approximately six hours after the surgery in the form of subcutaneous low-molecular-weight heparin (enoxaparin 40 mg). Paracetamol (1 g) infusion was given to all patients postoperatively. Additional analgesia in the form of a slow epidural infusion containing 0.5% bupivacaine (15 mL), 100 µg fentanyl (2 mL), and 0.9% normal saline (33 mL) was given only to patients with severe postoperative pain [17], and this was recorded. The amount of blood (in milliliters) in the drain was noted on the first postoperative day before drain removal. Drains were removed within 24 hours of surgery.

Quadriceps exercises, range of motion exercises, and protected weight-bearing ambulation was expected to start in the majority of the patients from the first postoperative day.

Postoperative pain was measured via the Visual Analog Scale (VAS) [17] every three hours for the first 24 hours, followed by 48 hours and at 72 hours postoperatively. Hemoglobin levels, hematocrit drift, and blood transfusion rates were measured and compared between the two groups preoperatively and then at 24 hours postoperatively.

The transfusion trigger was set at a postoperative blood hemoglobin value of less than 8.5 g/dL based on previous studies [15,18]. The time of starting postoperative knee mobilization exercises and weight-bearing was noted in all patients along with the duration of hospital stay. Immediate postoperative complications were noted in all patients.

Statistical analysis

Statistical analysis was done using SPSS version 21 (IBM Corp., Armonk, NY, USA). To determine whether the data were normally distributed, a Kolmogorov-Smirnov test was used. Comparison of quantitative variables between the study groups was done using the Mann-Whitney U test for independent samples for non-parametric data. For comparing categorical data, the chi-square test was performed, and the exact test was used when the frequency was less than 5. P-values of <0.05 were considered statistically significant.

## Results

Group A had 22 patients (28 knees), and group B had 20 patients (28 knees). The average age in our study was 64.04 years (range = 49 to 78 years). The majority of the patients were in the age group of 61-65 years. Overall, 76% of the patients (n = 32) were females. In total, 56 knees with osteoarthritis in 42 patients underwent TKA. The majority of knees were left-sided (n = 32), and 24 knees were right-sided. Group A had six patients undergoing bilateral TKA, while group B had nine patients undergoing the bilateral procedure.

**Figure 4:**
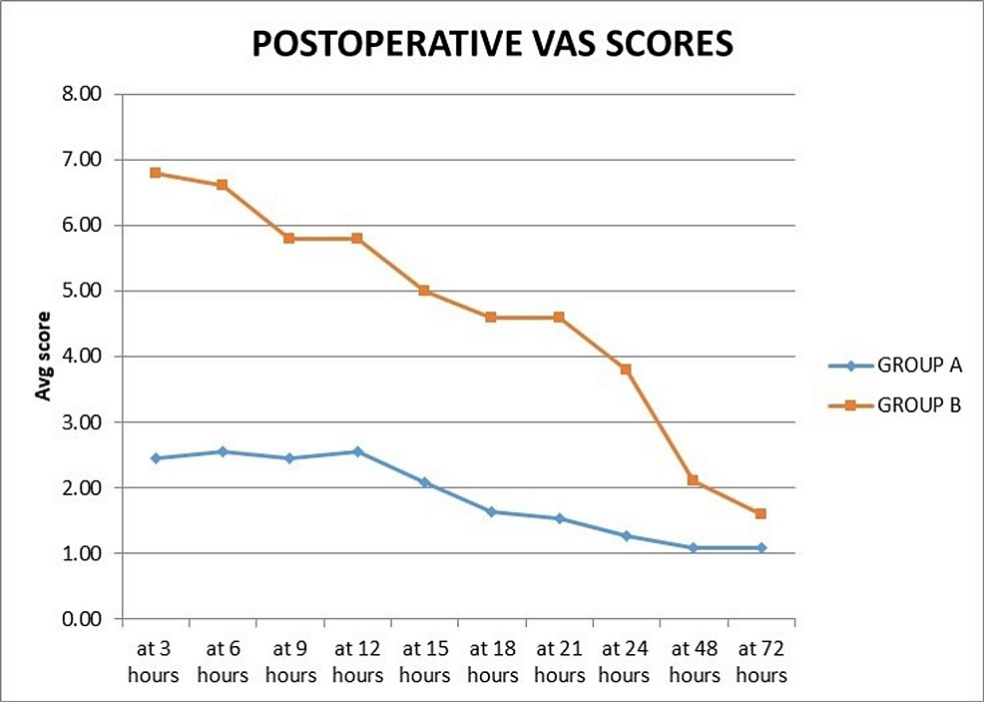
Graph showing the comparison of the average postoperative VAS scores at different hours. VAS: Visual Analog Scale

In group A, the mean VAS score at all times in the first 12 hours was ≤2.55. It decreased from 2.09 at 15 hours to 1.27 at 24 hours postoperatively. The mean VAS score was 1.09 at 48 hours and 72 hours. In Group B, in the first 12 hours, the mean VAS score was ≥5.8 at all times. It decreased from 5 at 15 hours to 3.8 at 24 hours. The mean VAS score at 48 hours was 2.10 and at 72 hours was 1.60. There was a statistically significant difference between VAS scores at all compared hours, except at 72 hours (Table 1, Figure 4).

**Table 1:** Comparison of the postoperative VAS scores. VAS: Visual Analog Scale; SD: standard deviation

Duration	Group A		Group B		Z-value	P-value
	Mean	SD	Mean	SD		
3 hours	2.45	0.69	6.80	1.69	-3.948	0.000
6 hours	2.55	0.82	6.60	1.65	-3.928	0.000
9 hours	2.45	1.21	5.80	1.14	-3.744	0.000
12 hours	2.55	2.50	5.80	1.48	-3.288	0.001
15 hours	2.09	1.38	5.00	1.05	-3.477	0.001
18 hours	1.64	0.67	4.60	0.97	-4.005	0.000
21 hours	1.55	0.69	4.60	0.97	-4.012	0.000
24 hours	1.27	0.65	3.80	1.48	-3.744	0.000
48 hours	1.09	0.30	2.10	1.37	-2.127	0.033
72 hours	1.09	0.30	1.60	0.97	-1.659	0.097

**Figure 5:**
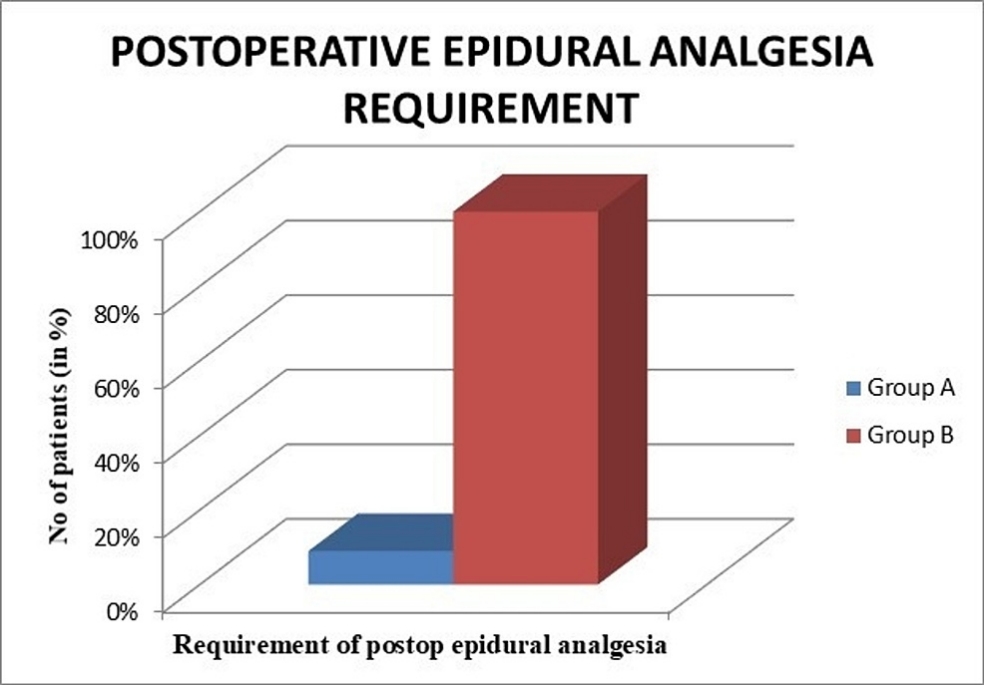
Graph showing the comparison of the requirement of postoperative epidural analgesia.

Group A had two (9%) patients who required epidural analgesia postoperatively. In group B, all 20 (100%) patients required epidural analgesia postoperatively. This difference was statistically highly significant (p = 0.0001) (Figure 5).

**Figure 6:**
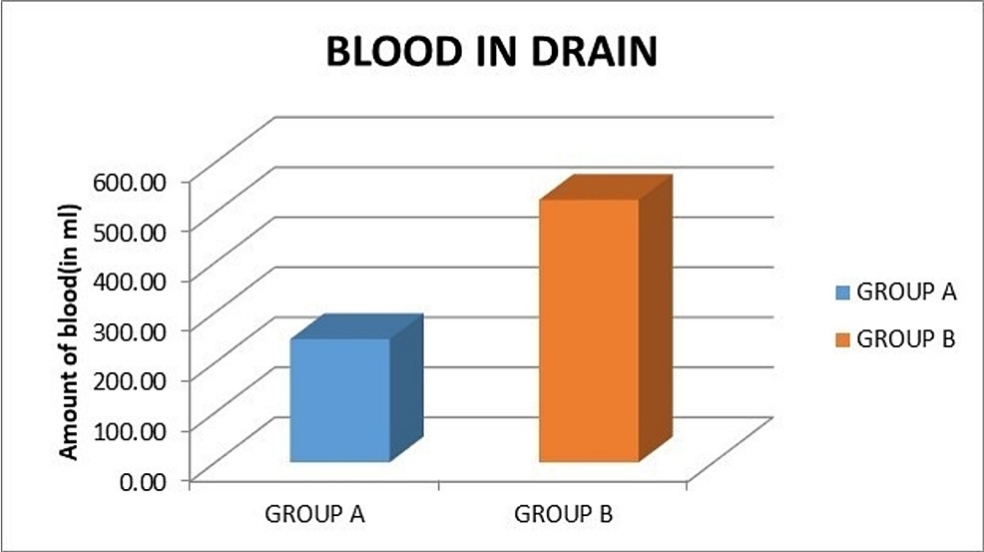
Graph showing the comparison of blood in the drain.

The mean amount of blood in the drain in group A (246.43 mL) was lower compared to group B (525 mL), which was statistically significant (p < 0.0001) (Figure 6).

**Figure 7:**
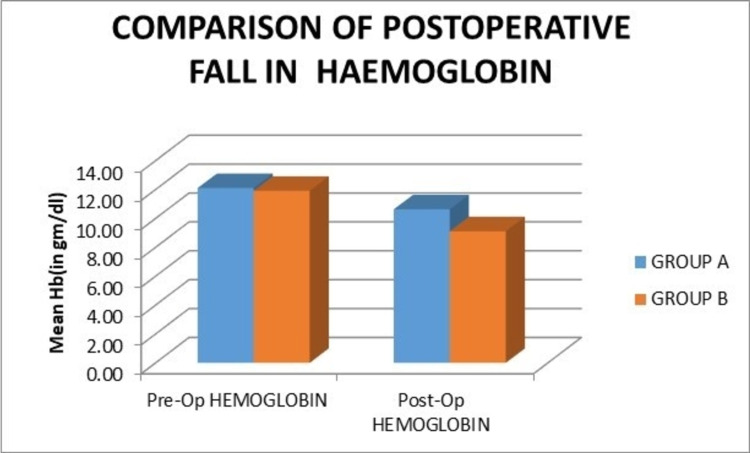
Graph showing the comparison of the postoperative fall in mean hemoglobin values. Pre-Op: preoperative; Post-Op: postoperative; Hb: hemoglobin

The mean preoperative hemoglobin in groups A and B was 12.10 g/dL and 11.91 g/dL, respectively. The mean postoperative hemoglobin in groups A and B was 10.63 g/dL and 9.11 g/dL, respectively. Moreover, the mean difference in preoperative and postoperative hemoglobin between groups A and B was 1.47 g/dL and 2.80 g/dL, respectively. There was a statistically significant difference in the postoperative hemoglobin values between the two groups at 24 hours. The fall in the hemoglobin levels after surgery was more in group B, and it was statistically significant when compared to that in group A (Figure 7).

**Figure 8:**
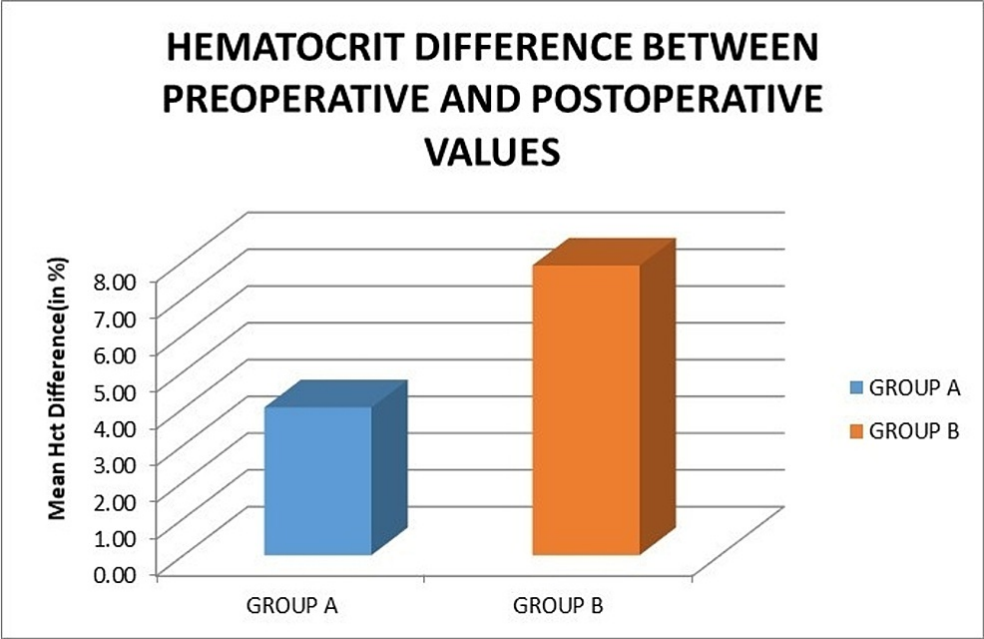
Graph showing the comparison of the postoperative fall in mean hematocrit values. Hct: hematocrit

The mean preoperative hematocrit in groups A and B was 36.34% and 35.46%, respectively. The mean postoperative hematocrit in groups A and B was 32.32% and 27.58%, respectively. Moreover, the mean difference in preoperative and postoperative hematocrit between groups A and B was 4.02% and 7.88%, respectively. There was a statistically significant difference between postoperative hematocrit fall among the two groups (Figure 8).

**Figure 9:**
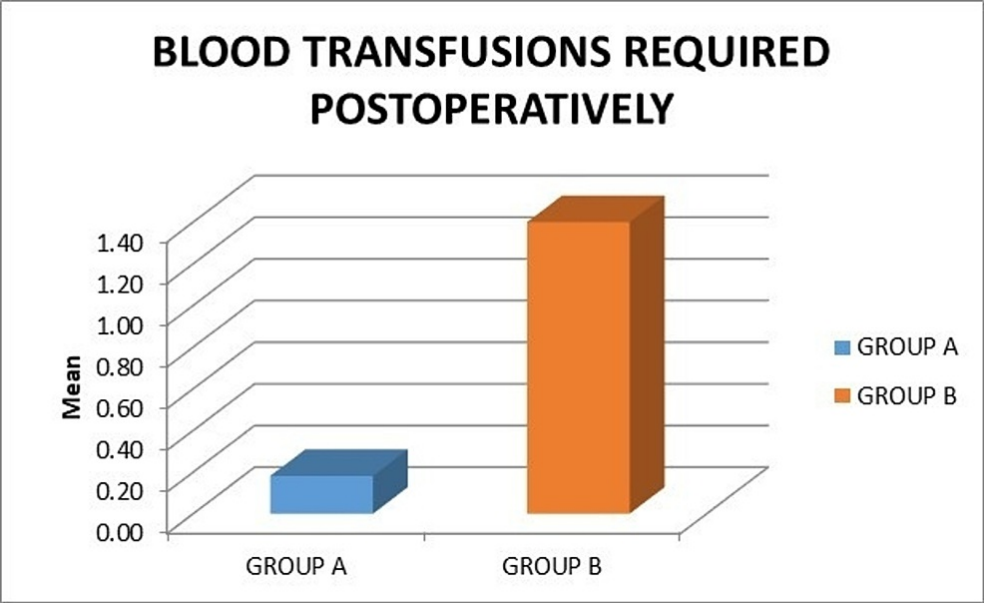
Graph showing the comparison of the mean postoperative blood transfusion requirement.

The mean number of blood transfusions required postoperatively in group A was 0.18, while in group B it was 1.40. This difference was statistically significant (p = 0.003) (Figure 9).

**Figure 10:**
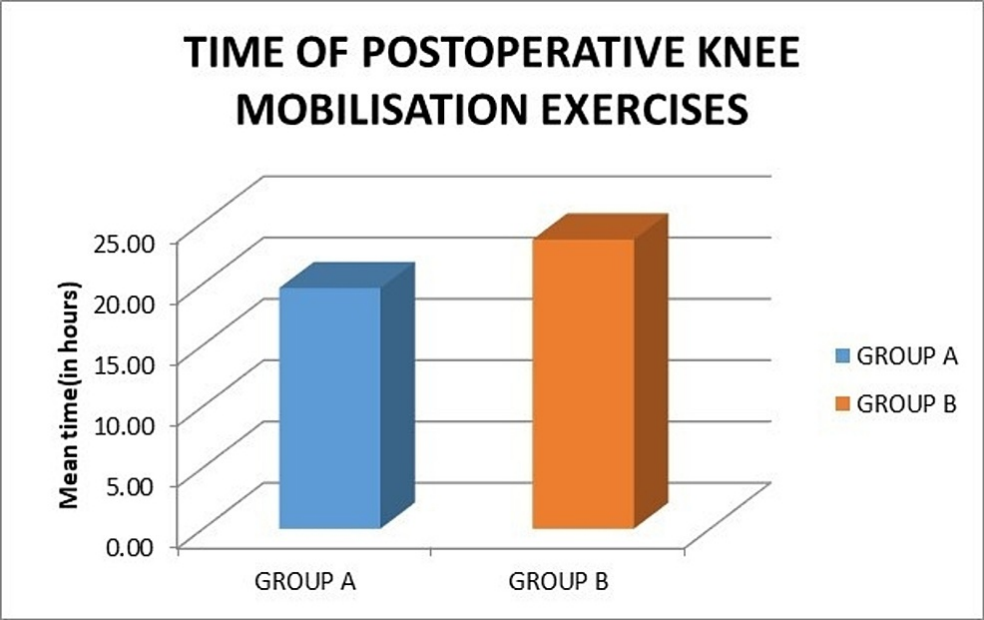
Graph showing the comparison of the mean time of postoperative knee mobilization exercises.

The mean time of postoperative knee mobilization exercises in group A was 19.64 hours, while in group B it was 23.60 hours, which did not show a statistically significant difference (p = 0.352) (Figure 10).

**Figure 11:**
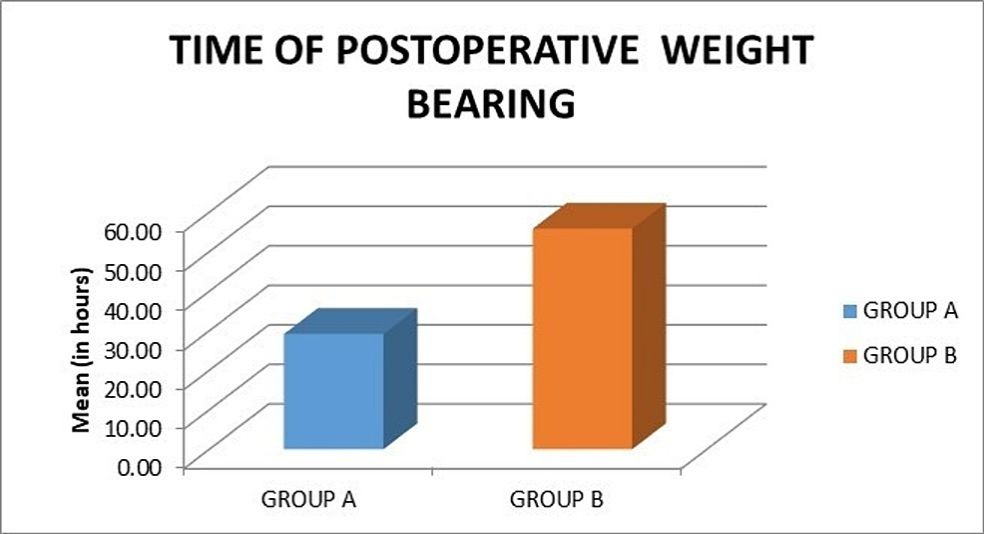
Graph showing the comparison of the mean time of postoperative weight-bearing.

The mean time of postoperative weight-bearing in group A was 29.18 hours, while in group B it was 55.80 hours. This difference was found to be statistically significant (p = 0.009) (Figure 11).

**Figure 12:**
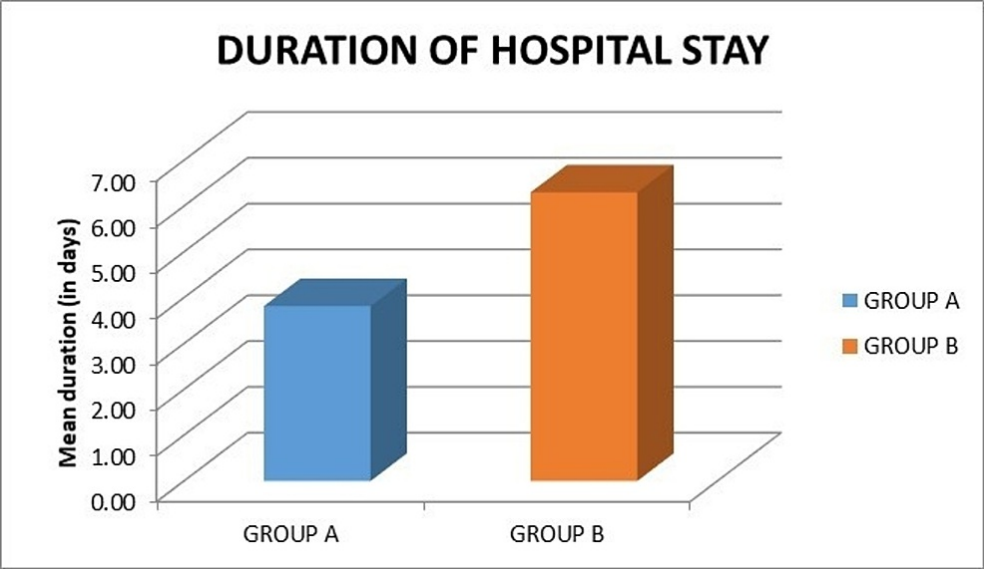
Graph showing the comparison of the mean duration of hospital stay.

In our study, the average duration of hospital stay was 3.82 days and 6.30 days, respectively, in groups A and B. This difference was statistically significant (p < 0.001) (Figure 12).

No major postoperative complications were noted in our study in both groups. Group B reported four cases of excessive postoperative pain (VAS score >8) and two cases of vomiting postoperatively. Table 2 presents the perioperative data.

**Table 2:** Perioperative data values.

Perioperative data values	Group A (n = 22)	Group B (n = 20)	P-value
Mean Blood in drain (in mL)	246.43 ± 95.97	525 ± 145.11	0.000 (significant)
Postoperative epidural analgesia required			
Yes	2 (9%)	20 (100%)	0.0001 (significant)
No	20 (91%)	0 (0%)	
Mean hemoglobin (in g/dL)			
Preoperative values	12.1 ± 1.00	11.91 ± 1.67	0.916 (not significant)
Postoperative values	10.63 ± 1.15	9.11 ± 1.65	0.020 (significant)
Hemoglobin fall values	1.47 ± 0.92	2.80 ± 1.29	0.005 (significant)
Mean hematocrit (in %)			
Preoperative values	36.34 ± 2.95	35.46 ± 5.12	0.888 (not significant)
Postoperative values	32.32 ± 3.11	27.58 ± 4.46	0.017 (significant)
Hematocrit fall values	4.02 ± 2.34	7.88 ± 3.85	0.007 (significant)
Mean postoperative blood transfusions	0.18 ± 0.60	1.40 ± 1.07	0.003 (significant)
Mean time of postoperative knee mobilization exercises (in hours)	19.64 ± 1.43	23.60 ± 8.25	0.352 (not significant)
Mean time of postoperative weight-bearing (in hours)	29.18 ± 11.20	55.80 ± 23.18	0.009 (significant)
Mean duration of hospital stay (in days)	3.82 ± 0.75	6.30 ± 1.70	0.000 (significant)

## Discussion

TKA has improved the quality of life of patients with knee arthritis. The number of knee replacements is increasing every year due to the increasing life expectancy and the consequent increase in the elderly population. Postoperative pain and blood loss are the two major disadvantages of TKA which affect patient satisfaction and hinder early rehabilitation. Further, increased bleeding after TKA may necessitate blood transfusion and result in knee joint swelling, restriction in knee motion, and delayed recovery. Many techniques and modalities are being tested in this regard. Intra-articular local infiltrations with local anesthetics such as ropivacaine have been shown to reduce initial severe postoperative pain [4,11,12,19]. The use of local antifibrinolytics such as tranexamic acid decreases the need for postoperative blood transfusions [5,13,15,18,20].

The average age in our study was 64.04 years, and the majority of patients were in the age group of 61 to 65 years. Pei et al. and Digas et al. reported a mean age of 65.5 years and 70 years, respectively, in their studies on TKA patients [20,15].

In our series, a female preponderance was seen, with 76% of the patients (n = 32) being females. In a study by Digas et al., there were more females compared to males (79 versus 11) [15]. In contrast to our study, Wang et al. found a male preponderance in their study (50 versus 40) on patients with osteoarthritis of the knee [14].

A left-sided preponderance was seen in our study in osteoarthritis patients (32 versus 24). Peng et al. reported right-sided preponderance, which was in contrast to our study (right: left = 55:38) [21].

In our study, in group A, the mean VAS score for pain assessment at all times in the first 12 hours was ≤2.55. It was 2.09 at 15 hours and decreased to 1.27 at 24 hours postoperatively. The mean VAS score was 1.09 at 48 hours and 72 hours. While in group B, in the first 12 hours, the VAS score was ≥5.8 at all times. It was 5 at 15 hours and decreased to 3.8 at 24 hours. The mean VAS score at 48 hours was 2.10 and at 72 hours was 1.60 in group B. Compared with the control knee, a statistically significant reduction in the mean VAS score was noted in the cocktail infiltrated knee until the first 24 hours (p < 0.001) and at 48 hours (p = 0.033). However, the difference in the mean VAS scores between both knees at 72 hours was not significant statistically (p = 0.097). There was statistically significant lower postoperative VAS score for pain, during the first 48 hours, in studies conducted by Vendittoli et al. [11] (p = 0.01), Pei et al. [20] (p = 0.001), and Kelly et al. [12] (p = 0.023).

In our series, group A had only two patients who required epidural analgesia (rescue analgesia) postoperatively. In group B, all 20 patients required epidural analgesia (rescue analgesia) postoperatively. These figures showed a statistically significant difference (p = 0.0001). In 2006, Vendittoli et al. showed that patient-controlled morphine consumption via an anesthesia pump was significantly lower in the local analgesia group in the first 48 hours compared to the control group (p = 0.0003) [11]. Similar results were obtained by Busch et al. in which patients who received the drug infiltration used significantly less patient-controlled analgesia compared to the controls (p < 0.001) [6].

In our series, the mean amount of blood in the drain in group A was lower compared to group B (246.43 mL versus 525 mL). There was a significant statistical difference in the amount of blood in the drain between the two groups (p < 0.0001). Similar results were obtained from various studies which reported statistically significant values [5,14,15,22].

In our series, the mean preoperative hemoglobin in groups A and B was 12.10 g/dL and 11.91 g/dL, respectively. The mean postoperative hemoglobin in these groups was 10.63 g/dL and 9.11 g/dL, respectively. Thus, the postoperative fall in hemoglobin levels at 24 hours in groups A and B was 1.47 g/dL and 2.80 g/dL, respectively. This difference between the postoperative fall of hemoglobin in the two groups was found to be statistically significant (p = 0.005). Similarly, a study in 2014 by Mutsuzaki et al. showed a mean difference between preoperative and postoperative hemoglobin in the control and the other group with intra-articular tranexamic acid infiltration to be 2.0 and 1.5, respectively, which was statistically significant (p < 0.024) [18].

In our study, the mean preoperative hematocrit in groups A and B was 36.34% and 35.46%, respectively. The mean postoperative hematocrit in these groups was 32.32% and 27.58%, respectively. The fall in hematocrit in groups A and B was 4.02% and 7.88%, respectively. There was a statistically significant difference in the postoperative hematocrit values (p = 0.017) between the two groups, although the preoperative hematocrit values in both groups were comparable. Further, the amount of postoperative fall of hematocrit was significantly less in group A compared to group B (p = 0.007). Similarly, a study in 2021 by Peng et al. showed a mean difference between preoperative and postoperative hematocrit in the control and peri-articular tranexamic acid injection group to be 9.73 and 7.39, respectively, which was statistically significant (p < 0.001) [21]. Volquind et al. showed a mean difference in preoperative and postoperative hematocrit in the control and intravenous tranexamic acid injection group of 10.81 and 8.84, respectively, which was statistically insignificant (p = 0.925) [22].

In our series, the mean postoperative blood transfusions required in group A was 0.18 and in group B was 1.40. Overall, 9.09% of patients required postoperative blood transfusion in group A, while 80% of patients required postoperative blood transfusion in group B. There was a statistically significant difference in the postoperative blood transfusion rates between the two groups (p = 0.003). Mutsuzaki et al. observed a similar result wherein the postoperative transfusion rates in patients receiving tranexamic acid via a drain to the joint was less compared to the control group (24.0% versus 60%) (p = 0.006) [18]. Studies by Seol et al. in 2016 and Digas et al. in 2021 showed similarly statistically significant differences between the two groups [5,15].

The mean time of postoperative knee mobilization exercises in group A was 19.64 hours, while in group B was 23.60 hours, which did not show a statistically significant difference (p = 0.352). In contrast to our study, Parvataneni et al. showed early straight leg raise and early knee motion in peri-articular injection compared to controls which was statistically significant (p = 0.05) [3].

The mean time of postoperative weight-bearing in group A was 29.18 hours, while in group B was 55.80 hours. There was a statistically significant difference in these values between the compared groups (p = 0.009).

In our study, the mean duration of hospital stay was 3.82 and 6.30 days, respectively, in groups A and B, and this difference was statistically significant (p < 0.001). This was similar to a study by Pei et al. where the average hospital stay was 7.1 days in the group that received intra-articular injection compared to 8.7 days in the control group (p = 0.003) [20]. However, in the series by Parvataneni et al., the mean hospitalization was 3.2 days in both groups receiving a peri-articular injection of 0.5% bupivacaine and the control group, which was found to be statistically insignificant (p > 0.05) [3].

The local infiltration technique used in our study had the benefits of low cost, high effectiveness, no systemic side effects, and ease of use. This combination of local infiltration of ropivacaine cocktail with tranexamic acid instillation reported good results in terms of postoperative pain relief and blood loss control. This method does not need any special training, unlike techniques such as epidural anesthesia or femoral nerve blocks, which require experience and further training.

## Conclusions

Patients with intra-articular infiltration using ropivacaine cocktail and tranexamic acid in TKA patients showed excellent pain control over non-infiltrated patients in the early 48 hours postoperatively. The intra-articular infiltration drastically reduced the requirement of rescue epidural analgesia immediately in the postoperative period, thus enabling early mobilization and weight-bearing. There was significantly less blood loss seen in the study group in which the infiltration was done, thus reducing the requirement of postoperative blood transfusions as well as the duration of hospital stay. Thus, it could be safely concluded that this technique of ropivacaine cocktail and tranexamic acid instillation postoperatively in knee arthroplasty patients is a very useful and effective technique to reduce postoperative pain and blood loss and to hasten recovery and rehabilitation.
